# 

**Published:** 1947

**Authors:** 


### CONTENTS.

| | Page |
|---|---|
| War Injuries of the Face and Jaws. By G. M. FitzGibbon, M.D., F.R.C.S. | 61 |
| The Rhesus Factors—I: Serology. By Geoffrey H. Tovey, M.D. | 68 |
| The Rhesus Factors—II: Haemolytic Disease of the New-Born. By Beryl D. Corner, M.D., B.S., M.R.C.P. | 74 |
| Reviews of Books | 81 |
| Editorial Note | 85 |
| Meetings of Societies | 86 |
| The Medical Library of the University of Bristol | 86 |
| Publications Received | 89 |
| Local Medical Notes | 90 |

